# Honokiol Ameliorates Myocardial Ischemia/Reperfusion Injury in Type 1 Diabetic Rats by Reducing Oxidative Stress and Apoptosis through Activating the SIRT1-Nrf2 Signaling Pathway

**DOI:** 10.1155/2018/3159801

**Published:** 2018-02-20

**Authors:** Bin Zhang, Mengen Zhai, Buying Li, Zhenhua Liu, Kaifeng Li, Liqing Jiang, Meng Zhang, Wei Yi, Jian Yang, Dinghua Yi, Hongliang Liang, Zhenxiao Jin, Weixun Duan, Shiqiang Yu

**Affiliations:** ^1^Department of Cardiovascular Surgery, Xijing Hospital, The Fourth Military Medical University, 127 Changle West Road, Xi'an 710032, China; ^2^Institute of Material Medical, School of Pharmacy, The Fourth Military Medical University, 169 Changle West Road, Xi'an 710032, China

## Abstract

Reducing oxidative stress is a crucial therapeutic strategy for ameliorating diabetic myocardial ischemia/reperfusion (MI/R) injury. Honokiol (HKL) acts as an effective cardioprotective agent for its strong antioxidative activity. However, its roles and underlying mechanisms against MI/R injury in type 1 diabetes (T1D) remain unknown. Since SIRT1 and Nrf2 are pivotal regulators in diabetes mellitus patients suffering from MI/R injury, we hypothesized that HKL ameliorates diabetic MI/R injury via the SIRT1-Nrf2 signaling pathway. Streptozotocin-induced T1D rats and high-glucose-treated H9c2 cells were exposed to HKL, with or without administration of the SIRT1 inhibitor EX527, SIRT1 siRNA, or Nrf2 siRNA, and then subjected to I/R operation. We found that HKL markedly improved the postischemic cardiac function, decreased the infarct size, reduced the myocardial apoptosis, and diminished the reactive oxygen species generation. Intriguingly, HKL remarkably activated SIRT1 signaling, enhanced Nrf2 nuclear translocation, increased antioxidative signaling, and decreased apoptotic signaling. However, these effects were largely abolished by EX527 or SIRT1 siRNA. Additionally, our cellular experiments showed that Nrf2 siRNA blunted the cytoprotective effects of HKL, without affecting SIRT1 expression and activity. Collectively, these novel findings indicate that HKL abates MI/R injury in T1D by ameliorating myocardial oxidative damage and apoptosis via the SIRT1-Nrf2 signaling pathway.

## 1. Introduction

Coronary artery disease, such as myocardial ischemia/reperfusion (MI/R) injury, predominates outcomes in patients with type 1 diabetes (T1D) with higher overall mortality rates compared to those of nondiabetes [1, 2]. Although timely reperfusion is necessary for salvaging the ischemic myocardium, it simultaneously induces a burst of reactive oxygen species (ROS) production and mitochondrial dysfunction, resulting in cardiomyocyte apoptosis and necrosis and ultimately destroying cardiac contractile function [3, 4]. In addition, accumulating evidence has indicated that prolonged hyperglycemia in the diabetic state can dramatically increase ROS accumulation, thereby aggravating the oxidative stress and apoptosis of myocardium during MI/R injury [5], which may explain the poor prognosis in diabetic patients after MI/R insult. At present, no drugs are being tested in clinical trials that can clearly abate MI/R injury in patients with T1D. Therefore, an urgent need exists to explore new therapies and elucidate their protective mechanisms.

Honokiol (HKL, C_18_H_18_O_2_, CAS number: 35354-74-6), a natural biphenolic compound derived from *Magnolia grandiflora* seed cone extract, has been widely used in traditional Chinese medicine [6]. Numerous findings imply that the beneficial activities of HKL can be attributed to a large extent to its antioxidative property [7, 8]. Intriguingly, HKL has shown a solid protective action against I/R injury in the ovaries, kidneys, brain, and heart [9–12]. Additionally, previous literatures demonstrate that HKL also exerts salutary metabolic effects in diabetic animal models [13, 14]. However, whether HKL administration could protect against MI/R injury in T1D and the underlying mechanisms remain unknown.

Silent information regulator l (SIRT1), a nicotinamide adenosine dinucleotide- (NAD^+^-) dependent deacetylase, has been indicated to affect multiple cellular processes and exert great influence in tissue injury and repair [15, 16]. Previously, we reported that SIRT1 activation played significant functions in ameliorating MI/R injury in diabetic models [17, 18]. Of interest, Avtanski et al. found that SIRT1 is involved in the beneficial effects of HKL in antagonizing the oncogenic actions of leptin in breast cancer [19]. Therefore, we hypothesized that SIRT1 signaling might mediate the protective effects of HKL against MI/R injury in T1D. Recently, it has been reported that nuclear factor-erythroid 2-related factor 2 (Nrf2), a vital antioxidant sensor for cellular defense mechanisms, served as an important downstream target of SIRT1 signaling in increasing resistance to oxidative damage [20, 21]. Moreover, Rajgopal et al. found that HKL could stimulate the Nrf2 pathway in hepatocytes and protect against oxidative stress [22]. However, whether Nrf2 could be modulated by HKL in MI/R-injured diabetic myocardium and its specific relationship with SIRT1 signaling in this circumstance remain elusive.

Based on the above observations, the aims of the present study were to evaluate the protective actions of HKL treatment against MI/R injury in diabetic settings and determine whether the SIRT1-Nrf2 signaling plays a regulatory role in this process both *in vivo* and *in vitro*.

## 2. Materials and Methods

### 2.1. Animals

All procedures were performed in accordance with the *Guide for the Care and Use of Laboratory Animals* by the National Academy of Sciences and published by the National Institutes of Health (NIH Publication No. 86-23, revised 1996). The Institutional Animal Care and Use Committee at Xijing Hospital, the Fourth Military Medical University, reviewed and approved the protocol. Sprague-Dawley (SD) rats, weighing 180–220 g at 8 weeks of age, were obtained from the experimental animal center of the Fourth Military Medical University. Animals were housed at 22°C with a 12 h light/dark cycle and given free access to water and a standard diet.

### 2.2. Reagents

HKL and EX527 were obtained from Sigma-Aldrich (MO, USA). The terminal deoxynucleotidyl transferase-mediated dUTP nick end labeling (TUNEL) assay kit was purchased from Roche Biochemicals (Mannheim, Germany). Kits for detecting lactate dehydrogenase (LDH), creatine kinase (CK), superoxide dismutase (SOD) activities, and malondialdehyde (MDA) content were purchased from the Institute of Nanjing Jiancheng Bio-Engineering Institute (Nanjing, Jiangsu, China). A primary antibody against gp91*^phox^* was obtained from Santa Cruz Biotechnology (CA, USA). Primary antibodies against cleaved caspase 3, Bcl-2, Bax, cytochrome *c*, and *β*-actin were all purchased from Cell Signaling Technology (Boston, MA, USA). Primary antibodies against SIRT1, Nrf2, heme oxygenase-1 (HO-1), NAD(P)H quinone oxidoreductase 1 (NQO-1), and histone H3 were purchased from Abcam (Cambridge, MA, USA). Rabbit antigoat, goat antimouse, and goat antirabbit secondary antibodies were obtained from the Zhongshan Company (Beijing, China).

### 2.3. Establishment of a T1D Rat Model

A T1D rat model was constructed as previously described [23, 24]. SD rats were fasted overnight and received an intraperitoneal injection of streptozotocin (STZ; Sigma-Aldrich, MO, USA) dissolved in sterile citrate buffer (0.1 mM, pH 4.5, 60 mg/kg/d) for 3 days. Control rats received only citrate buffer injections. Seven days after the last injection, the rats with fasting plasma glucose (PG) levels of >11.1 mM were defined as diabetic and housed for another 14 days. Next, the oral glucose tolerance test (OGTT) and intraperitoneal glucose tolerance test (IPGTT) were conducted to further confirm the successful establishment of the T1D rat model. Blood glucose levels were measured before glucose loading (0 min), and 30, 60, 90, and 120 min after glucose loading.

### 2.4. MI/R Protocol

The MI/R injury model was generated and utilized as previously described [25, 26]. Rats were anesthetized using an intraperitoneal injection of pentobarbital sodium (50 mg/kg) and ventilated via tracheal intubation with a Harvard rodent respirator. Body temperature was maintained at 37°C via a heated operating table. The chest was opened by a left lateral thoracotomy, and the left anterior descending coronary artery was ligated at approximately 2 mm distal to its origin from the coronary artery occlusion. MI/R injury was induced by ischemia for 30 min, followed by reperfusion for 4 h. Ischemia was confirmed by noting the change in color of myocardial tissue in the ischemic area, and reperfusion was achieved by loosening the knot. In sham-operated rats, the suture was passed but not tied. The animals were randomly assigned to the following groups: (1) Con + sham group; (2) Con + MI/R group; (3) T1D + sham group; (4) T1D + MI/R group; (5) T1D + MI/R + HKL group; and (6) T1D + MI/R + HKL + EX527 group. HKL (5 mg/kg/d, diluted in sterile saline containing less than 1% dimethyl sulfoxide (DMSO)) was orally administered for 1 week before the surgery and intraperitoneally injected once 10 min before the reperfusion. EX527 (5 mg/kg/d, diluted in sterile saline containing less than 1% DMSO) was intraperitoneally administered once daily for 3 days before the surgery and once 20 min before the reperfusion. The experimental animal scheme was illustrated in Figure S1, and the dosages of HKL and EX527 were based on previous studies [12, 14, 18, 25].

### 2.5. Echocardiography

Transthoracic echocardiography was performed to measure cardiac function with a Visualsonics Vevo 770 echocardiography machine (Toronto, Ontario, Canada), as described previously [17]. Briefly, rats were anesthetized with isoflurane 72 h after the MI/R operation, and motion- (M-) mode echocardiographic images were obtained at the level of the papillary muscles to measure the left ventricular ejection fraction (LVEF) and left ventricular fractional shortening (LVFS).

### 2.6. Myocardial Infarct Sizes

Following reperfusion, the artery was reoccluded, and 2% Evans blue dye was injected into the left ventricular cavity and allowed to perfuse the nonischemic portions of the myocardium. Then the heart was sectioned horizontally into six slices (1 mm thick) from the apex. The red-stained areas-at-risk (AAR) were dissected after the slices were immersed in 2,3,5-triphenyltetrazolium chloride (TTC; 1% in PBS, Sigma-Aldrich, MO, USA) at 37°C for 20 min. The infarcted tissue was stained white or light yellow. After fixation in 4% paraformaldehyde solution, each side of the stained tissue slices was digitally scanned. The infarct size was determined as the percentage of AAR, using Image-Pro Plus software (Media Cybernetics, MD, USA), as described previously [27].

### 2.7. Evaluation of LDH Release and CK Activity, and Quantification of SOD and MDA

Myocardial necrosis was estimated based on serum LDH and CK activities, using kits from the Institute of Nanjing Jiancheng Bio-Engineering Institute, per the manufacturer's instructions. The oxidative stress markers, SOD activity and MDA content, were determined spectrophotometrically with a SpectraMax M5 instrument (Molecular Devices, CA, USA), using commercially available kits, following the manufacturer's instructions.

### 2.8. TUNEL Assay

Apoptosis was analyzed by performing TUNEL assays using the In Situ Cell Death Detection Kit according to the manufacturer's instructions. A double-staining technique was used: TUNEL-positive cells displayed green fluorescence, and all nuclei stained with DAPI produced blue fluorescence. The apoptotic index was expressed as the number of positively stained apoptotic cardiomyocytes divided by the total number of cardiomyocytes.

### 2.9. Cell Culture and *In Vitro* Experiments

Rat embryonic cardiomyoblast-derived H9c2 cells (Tiancheng Technology, Shanghai, China) were used to study MI/R *in vitro* and were cultured in Dulbecco's modified Eagle's medium (DMEM; HyClone, Logan, UT, USA) containing 10% fetal bovine serum and penicillin/streptomycin solution in a humidified cell culture incubator (21% O_2_, 5% CO_2_, 37°C). Cells at passages 4–6 were grown until confluence for experiments. Simulated ischemia-reperfusion (SIR) injury was initiated by incubating H9c2 cells for 1 h in an ischemic buffer (pH 6.5) containing 137 mM NaCl, 0.49 mM MgCl_2_, 12 mM KCl, 0.9 mM CaCl_2_, 4 mM HEPES, 0.75 mM sodium dithionate, 10 mM deoxyglucose, and 20 mM lactate. Reperfusion was accomplished by exchanging the buffer with serum-free DMEM and incubating for another 4 h.

The pathophysiological conditions of the diabetic state were replicated *in vitro*, as described previously [28–30]. H9c2 cells were cultured in high-glucose (HG) medium (33 mM glucose) for 8 h before SIR treatment and during the entire reperfusion period to mimic the *in vivo* diabetic model. Normal-glucose (NG) medium containing 5.5 mM glucose was used as a control (supplemented with 27.5 mM mannitol to adjust the total osmotic pressure to 33 mM). HKL was coadministered with HG to evaluate its cytoprotective effects.

### 2.10. Cell Viability

Cultured H9c2 cells were seeded in 96-well plastic plates. Cell viability was assessed by cell-counting kit-8 (CCK-8) assay according to the manufacturer's instructions and presented by dividing the optical density of samples with that of the HG group. Briefly, CCK-8 solution was added to each well of 10 *μ*L after reperfusion and incubated in the dark for 2 h at 37°C before measuring. Absorbance at 450 nm was read spectrophotometrically using a microplate reader.

### 2.11. Small-Interfering RNA (siRNA) Transfections

Predesigned and validated siRNAs specific for SIRT1, Nrf2, and a nontargeting control were purchased from GenePharma (Suzhou, China). The special sequences of SIRT1 siRNA were sense: 5′-CCA GUA GCA CUA AUU CCA ATT-3′, antisense: 5′-UUG GAA UUA GUG CCA CUG GTT-3′. The special sequences of Nrf2 siRNA were sense: 5′-GAG GAU GGG AAA CCU UAC UTT-3′, antisense: 5′-AGU AAG GUU UCC CAU CCU CTT-3′. The special sequences of negative control were sense: 5′-UUC UCC GAA CGU GUC ACG UTT-3′, antisense: 5′-ACG UGA CAC GUU CGG AGA ATT-3′. H9c2 cells were plated to reach 70–90% confluency on the day of transfection. Each siRNA duplex was introduced with the Lipofectamine 3000 reagent for 24 h (Invitrogen, Carlsbad, CA, USA) in Opti-MEM medium (Gibco, Carlsbad, CA, USA).

### 2.12. ROS Detection

The presence of ROS in myocardial frozen sections was detected using the oxidative fluorescent dye dihydroethidium (DHE; Sigma-Aldrich, MO, USA). Each heart was perfusion-fixed with 4% paraformaldehyde, 5% sucrose, and 20 mM EDTA (pH 7.4) for 10 min; then excised and embedded in optimal-cutting-temperature compound; flash frozen in liquid nitrogen; and stored at −80°C. Serial 10-*μ*m-thick sections of each heart were stained with DHE according to the manufacturer's instructions. Cellular mitochondrial ROS production was measured using a fluorescent microplate reader with 2′,7′-dichlorofluorescin diacetate (DCFDA; Beyotime Biotechnology, Shanghai, China), which is de-esterified intracellularly and is converted to the highly fluorescent molecule 2′,7′-dichlorofluorescin (DCF) in the presence of ROS. Mitochondrial ROS levels were determined as the intensity of DCF fluorescence at an excitation wavelength of 488 nm and an emission wavelength of 525 nm.

### 2.13. Immunofluorescence Staining

The cultured cells were rinsed with PBS, blocked with goat serum, incubated with a primary anti-SIRT1 antibody at 4°C overnight, and subsequently incubated with a Cy3-conjugated goat antimouse IgG for 2 h at room temperature. After the cells were washed three times in PBS, they were counterstained with DAPI for nucleus identification. Images were captured digitally using an Olympus FV10C-W3 laser confocal microscope (Olympus, Japan) in five random fields for each sample.

### 2.14. SIRT1 Activity Measurements

SIRT1 deacetylase activity was measured using a fluorometric assay (Enzo Life Sciences, catalogue number: BML-AK555-0001) according to the manufacturer's instructions. After the Developer II was added to each well, the reaction mixture was incubated at 37°C for an additional 1 h. The fluorescence of each sample was read using a SpectraMax M5 instrument with an excitation set to 360 nm and emission set to 460 nm.

### 2.15. Western Blot Analysis

Cell and tissue lysate homogenates were prepared as previously described [25]. Nuclear extracts were prepared using a nuclear extraction kit (Sigma-Aldrich, MO, USA). After quantification with the BCA Protein Assay Kit, equal amounts of protein samples (40 *μ*g) were separated by 10% sodium dodecyl sulfate-polyacrylamide gel electrophoresis and transferred to a polyvinylidene fluoride membrane (Millipore, MA, USA). After being blocked in TBST buffer (50 mM Tris, 150 mM NaCl, 0.1% Tween 20, pH 7.6) containing 5% skim milk for 2 h at room temperature, the membranes were incubated with appropriate primary antibodies against SIRT1 (1 : 1000), Nrf2 (1 : 1000), NQO-1 (1 : 1000), HO-1 (1 : 1000), gp91*^phox^* (1 : 500), Bax (1 : 1000), Bcl-2 (1 : 1000), cytosolic cytochrome *c* (1 : 1000), cleaved caspase 3 (1 : 1000), histone H3 (1 : 1000), and *β*-actin (1 : 1000) overnight at 4°C. Then membranes were washed in TBST and reacted with a secondary horseradish peroxidase-conjugated antibody (1 : 5000) for 1.5 h at 37°C. Antigen-antibody complexes were then visualized using enhanced chemiluminescence reagents. The density of the immunoreactive bands was analyzed using Image Lab software (Bio-Rad Laboratories, CA, USA).

### 2.16. Statistical Analysis

Data are expressed as the mean ± SEM. The statistical significance of differences was determined by a Student *t* test between two groups or one-way ANOVA followed by Bonferroni's multiple-comparison for post hoc *t* test in GraphPad Prism software (version 5.0, GraphPad Software, San Diego, CA, USA). A *P* value less than 0.05 was considered statistically significant.

## 3. Results

### 3.1. T1D Rats Showed Exacerbated Cardiac Damage with Further Reduced Myocardial SIRT1 Expression and Activity following MI/R Injury

**Figure 1:**
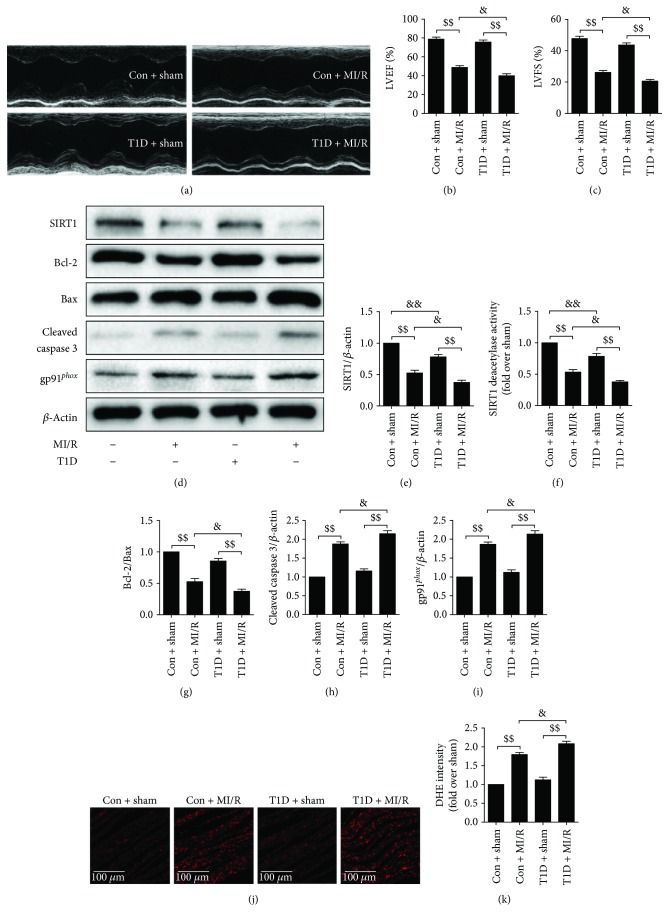
Type 1 diabetic rats subjected to MI/R surgery showed further weakened cardiac function, increased myocardial oxidative stress and apoptosis, and reduced myocardial SIRT1 signaling. (a) Representative M-mode images by echocardiography. Echocardiographic assessment was performed after 72 h of reperfusion. (b) Left ventricular ejection fraction (LVEF). (c) Left ventricular fractional shortening (LVFS). (d) Representative blots. (e) SIRT1 expression. (f) Relative SIRT1 activity. (g) Bcl-2/Bax ratio. (h) Cleaved caspase 3 expression. (i) gp91*^phox^* expression. (j) Representative images of DHE staining (300x, bar = 100 *μ*m). (k) DHE intensity. Data are presented as the mean ± SEM (*n* = 6 in each group). ^$$^ *P* < 0.01 versus the sham group, ^&/&&^ *P* < 0.05/0.01 versus the Con group.

As expected, in comparison to the Con group, T1D rats exhibited significantly increased levels of nonfasting and fasting PG, accompanied by markedly impaired IPGTT and OGTT, indicating that the T1D rat model was successfully established (Figure S2). In both diabetic and nondiabetic rats, cardiac functions were markedly impaired after MI/R insult, as confirmed by decreased LVEF and LVFS. However, T1D further aggravated these MI/R-induced cardiac damages (Figures 1(a)–1(c)). In addition, we found markedly increased expressions of apoptosis-related proteins, ROS production, and gp91*^phox^* expression in the T1D + MI/R group, suggesting that T1D exacerbated myocardial apoptosis and oxidative stress following MI/R operation (Figures 1(g)–1(k)). Furthermore, we observed dramatically decreased SIRT1 expression and activity in T1D rats, which was further attenuated following MI/R injury (Figures 1(e)-1(f)).

### 3.2. EX527 Abolished HKL-Induced Alleviation of Cardiac Damage in T1D following MI/R Injury

**Figure 2:**
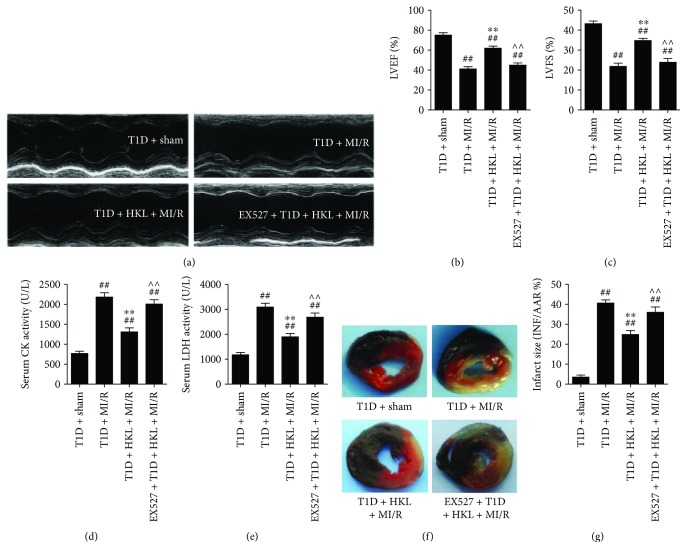
EX527 abolished HKL-induced cardioprotective effects on MI/R injury in type 1 diabetic rats. (a) Representative M-mode images by echocardiography. Echocardiographic assessment was performed after 72 h of reperfusion. (b) Left ventricular ejection fraction (LVEF). (c) Left ventricular fractional shortening (LVFS). (d) Serum CK activity. (e) Serum LDH activity. (f) Representative digital images of heart sections by Evans blue and triphenyltetrazolium chloride (TTC) double staining. (g) Myocardial infarct size. Data are presented as the mean ± SEM (*n* = 6 in each group). ^#/##^ *P* < 0.05/0.01 versus the T1D + sham group, ^∗/∗∗^ *P* < 0.05/0.01 versus the T1D + MI/R group, ^∧/∧∧^ *P* < 0.05/0.01 versus the T1D + MI/R + HKL group.

To explore the role of HKL on MI/R insult in T1D, as well as elucidating the underlying mechanisms, we employed the SIRT1 specific inhibitor EX527 in our *in vivo* experiments. As shown in Figures 2(a)–2(c), HKL notably improved postischemic cardiac function in T1D rats by increasing LVEF and LVFS. Moreover, MI/R injury was markedly alleviated with HKL supplementation, as evidenced by reduced myocardial infarction size and decreased serum CK and LDH activities (Figures 2(d)–2(g)). However, these cardioprotective activities were all blunted by EX527 administration. In addition, the experimental dosage of EX527 had no significant toxic effects on diabetic hearts (Figure S3). These data all suggested that HKL protected against MI/R injury in diabetic rats and that SIRT1 played a pivotal role in this process.

### 3.3. EX527 Blunted HKL-Induced Amelioration of the Oxidative Stress Level in Diabetic Myocardium following MI/R Injury

**Figure 3:**
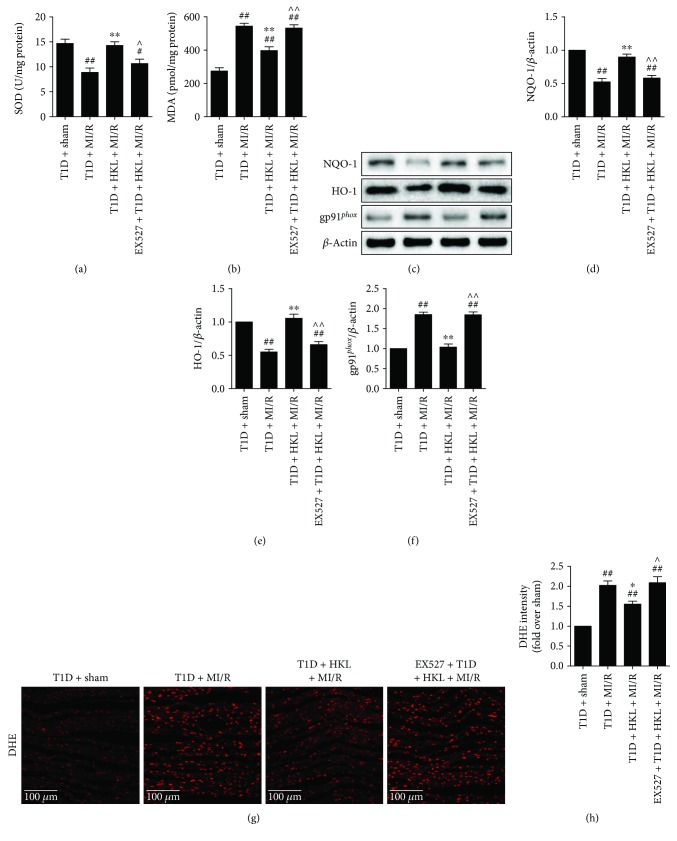
EX527 blunted HKL-induced suppression on oxidative stress level in MI/R-injured diabetic myocardium. (a) Myocardial SOD contents. (b) Myocardial MDA contents. (c) Representative blots. (d) NQO-1 expression. (e) HO-1 expression. (f) gp91*^phox^* expression. (g) Representative images of DHE staining (300x, bar = 100 *μ*m). (h) DHE intensity. Data are presented as the mean ± SEM (*n* = 6 in each group). ^#/##^ *P* < 0.05/0.01 versus the T1D + sham group, ^∗/∗∗^ *P* < 0.05/0.01 versus the T1D + MI/R group, ^∧/∧∧^ *P* < 0.05/0.01 versus the T1D + MI/R + HKL group.

As shown in Figures 3(a)-3(b) and 3(g)-3(h), HKL exhibited its antioxidative capacity by improving SOD activity and reducing ROS production and MDA generation. However, inhibiting SIRT1 signaling blunted these effects. Furthermore, the expression levels of two antioxidative molecules, HO-1 and NQO-1, and one oxidative stress marker, gp91*^phox^*, were assessed by western blot analysis. Compared with the T1D + MI/R group, HKL administration markedly upregulated HO-1 and NQO-1 expressions, while downregulating gp91*^phox^* expression (Figures 3(c)–3(f)). Consistently, EX527 administration abolished these effects. Collectively, these data indicated that SIRT1 participated in the ameliorative effect of HKL against myocardial oxidative stress in diabetic heart following MI/R injury.

### 3.4. EX527 Abolished HKL-Induced Suppression of Cardiomyocyte Apoptosis in Diabetic Myocardium following MI/R Operation

**Figure 4:**
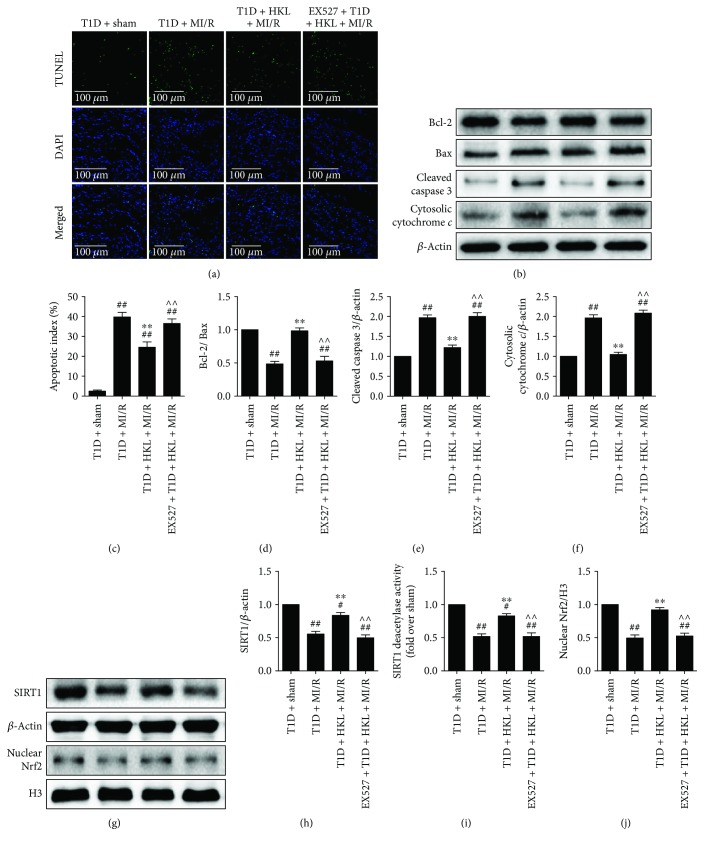
EX527 abolished HKL-induced suppression on myocardial apoptosis in MI/R-injured diabetic myocardium. (a) Representative images of TUNEL staining (300x, bar = 100 *μ*m). The apoptotic cells were detected by TUNEL (green), and the nuclei were detected by DAPI (blue). (b) Myocardial apoptotic index. (c) Representative blots. (d) Bcl-2/Bax ratio. (e) Cleaved caspase 3 expression. (f) Cytosolic cytochrome *c* expression. (g) Representative blots. (h) SIRT1 expression. (i) Relative SIRT1 activity. (j) Nrf2 nuclear translocation. Data are presented as the mean ± SEM (*n* = 6 in each group). ^#/##^ *P* < 0.05/0.01 versus the T1D + sham group, ^∗/∗∗^ *P* < 0.05/0.01 versus the T1D + MI/R group, ^∧/∧∧^ *P* < 0.05/0.01 versus the T1D + MI/R + HKL group.

Intriguingly, in our study, HKL showed a strong inhibitory effect against myocardial apoptosis in diabetic heart following MI/R operation, as confirmed by notably reduced apoptotic index, significantly decreased expression levels of cleaved caspase 3 and cytosolic cytochrome *c*, and remarkably increased ratio of Bcl-2/Bax (Figures 4(a)–4(f)). As expected, inhibition of SIRT1 signaling markedly blunted the antiapoptotic activity of HKL. These data suggested that the antiapoptotic capability of HKL against MI/R injury in T1D was regulated by SIRT1 signaling.

### 3.5. EX527 Attenuated HKL-Induced Increase of Myocardial SIRT1 Expression and Activity and Nrf2 Nuclear Translocation in MI/R-Injured Diabetic Myocardium

Notably, at the end of reperfusion, the expression and activity of SIRT1 were significantly increased by HKL treatment. As a marker of Nrf2 activation [31], the nuclear accumulation of Nrf2 was also markedly enhanced by HKL supplementation in comparison to the T1D + MI/R group. Interestingly, inhibiting myocardial SIRT1 signaling reversed these actions conferred by HKL (Figures 4(g)–4(j)).

### 3.6. SIRT1 siRNA and Nrf2 siRNA Transfection Blunted HKL-Induced Antiapoptotic Effects against SIR Injury in High-Glucose Medium-Cultured H9c2 Cells

**Figure 5:**
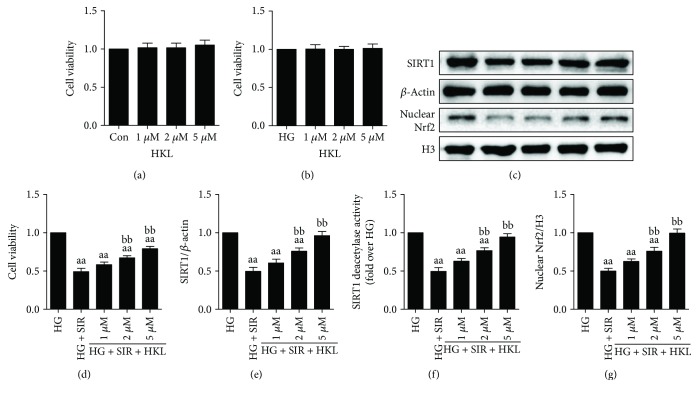
HKL treatment increased cell viability, raised intracellular SIRT1 expression and activity, and enhanced Nrf2 nuclear accumulation in HG-treated H9c2 cells after SIR injury. (a) HKL treatment (1 *μ*M, 2 *μ*M, and 5 *μ*M) had no significant effect on the cell viability of control H9c2 cells. (b) HKL treatment (1 *μ*M, 2 *μ*M, and 5 *μ*M) had no significant effect on the cell viability of HG-treated H9c2 cells. (c) Representative blots. (d) HKL treatment dose dependently increased the cell viability against SIR injury in HG-treated H9c2 cells. (e) SIRT1 expression. (f) Relative SIRT1 activity. (g) Nrf2 nuclear translocation. Data are presented as the mean ± SEM (*n* = 6 in each group). ^a/aa^ *P* < 0.05/0.01 versus the HG group, ^b/bb^ *P* < 0.05/0.01 versus the HG + SIR group.

To further confirm the cardioprotective mechanisms of HKL in T1D hearts, we performed *in vitro* experiments using H9c2 cardiomyoblasts. Initially, we found that high-glucose incubation significantly reduced the expression and activity of SIRT1, which were further attenuated after SIR injury (Figure S4(e)-4(f)). Consistent with our *in vivo* data, the hyperglycemic state exacerbated SIR-induced cellular oxidative stress and apoptosis (Figure S4). Intriguingly, as shown in Figure 5, HKL treatment alone, compared with the Con or HG group, had no toxic effect on cell survival, while it could significantly increase cell viability, SIRT1 expression and activity, and Nrf2 nuclear accumulation following SIR treatment in a dose-dependent manner. These phenomena were most obvious at an HKL concentration of 5 *μ*M, which was selected for further mechanism studies.

**Figure 6:**
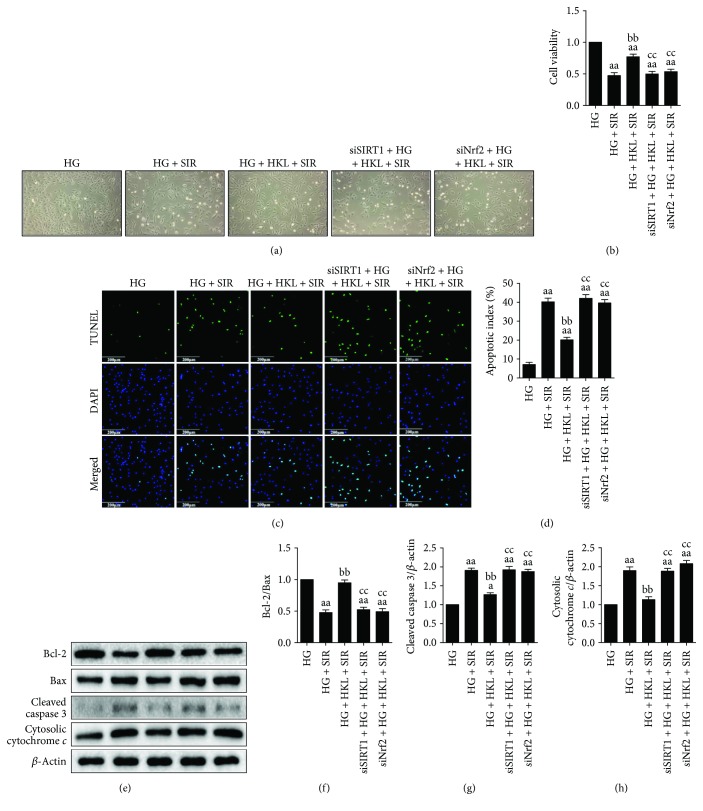
SIRT1 siRNA and Nrf2 siRNA transfection blunted HKL-induced antiapoptotic effects against SIR injury in HG-treated H9c2 cells. (a) Cellular morphology (100x). (b) Cellular viability. (c) Representative images of TUNEL staining (200x, bar = 200 *μ*m). The apoptotic cells were detected by TUNEL (green), and the nuclei were detected by DAPI (blue). (d) Cellular apoptotic index. (e) Representative blots. (f) Bcl-2/Bax ratio. (g) Cleaved caspase 3 expression. (h) Cytosolic cytochrome *c* expression. Data are presented as the mean ± SEM (*n* = 6 in each group). ^a/aa^ *P* < 0.05/0.01 versus the HG group, ^b/bb^ *P* < 0.05/0.01 versus the HG + SIR group, ^c/cc^ *P* < 0.05/0.01 versus the HG + SIR + HKL group.

Next, we determined the role of HKL on SIR-induced apoptosis in the hyperglycemic state. As shown in Figures 6(c)–6(h), HKL markedly reduced apoptosis by decreasing the percentage of TUNEL-positive nuclei, accompanied by an increased ratio of Bcl-2/Bax and reduced expressions of cleaved caspase 3 and cytosolic cytochrome *c*. In addition, HKL effectively improved cell viability and alleviated cell shrinking and detachment induced by SIR (Figures 6(a)-6(b)). Transfection with SIRT1 or Nrf2 siRNA significantly downregulated SIRT1 or Nrf2 expression, respectively, while the control siRNA had little effect on the cell viability, apoptosis, and oxidative stress to high-glucose-treated H9c2 cells (Figure S5). The cytoprotective effects of HKL were markedly blunted by SIRT1 siRNA or Nrf2 siRNA administration, indicating that SIRT1 and Nrf2 signaling are key mediators of the protective activities of HKL.

### 3.7. SIRT1 siRNA and Nrf2 siRNA Transfection Inhibited HKL-Induced Suppression of Oxidative Stress Level against SIR Injury in High-Glucose Medium-Cultured H9c2 Cells

**Figure 7:**
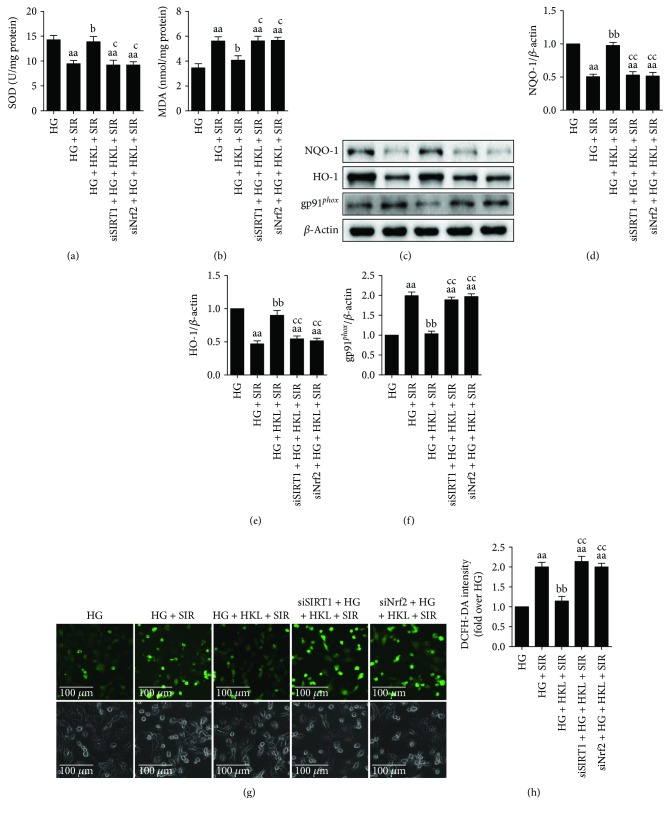
SIRT1 siRNA and Nrf2 siRNA transfection inhibited HKL-induced suppression on oxidative stress level against SIR injury in HG-treated H9c2 cells. (a) Intracellular SOD activity. (b) Intracellular MDA content. (c) Representative blots. (d) NQO-1 expression. (e) HO-1 expression. (f) gp91*^phox^* expression. (g) Representative images of DCFH-DA staining (300x, bar = 100 *μ*m). (h) DCFH-DA intensity. Data are presented as the mean ± SEM (*n* = 6 in each group). ^a/aa^ *P* < 0.05/0.01 versus the HG group, ^b/bb^ *P* < 0.05/0.01 versus the HG + SIR group, ^c/cc^ *P* < 0.05/0.01 versus the HG + SIR + HKL group.

In accordance with our *in vivo* data, HKL markedly increased SOD activity and decreased intracellular ROS production and MDA generation, whereas either SIRT1 siRNA or Nrf2 siRNA abolished these effects (Figures 7(a)-7(b) and 7(g)-7(h)). Simultaneously, western blot analysis showed that gp91*^phox^* expression significantly decreased in the HKL-treated group, accompanied by increased expressions of HO-1 and NQO-1. However, the effects were also blunted by SIRT1 siRNA or Nrf2 siRNA (Figures 7(c)–7(f)). These data showed that HKL could attenuate cellular oxidative damage against SIR injury via SIRT1 and Nrf2 signaling in high-glucose medium-cultured H9c2 cells.

### 3.8. SIRT1 Acted as an Upstream Regulator of Nrf2 in Mediating the Cardioprotective Actions of HKL

**Figure 8:**
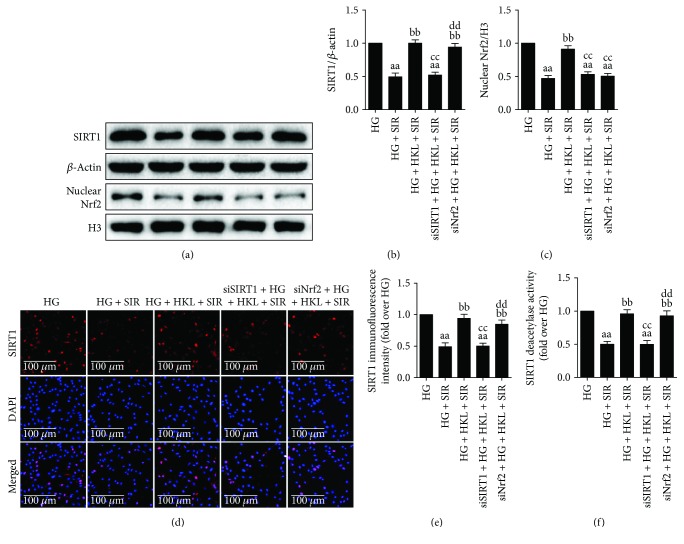
SIRT1 acted as an upstream regulator of Nrf2 in mediating the cardioprotective actions of HKL. (a) Representative blots. (b) SIRT1 expression. (c) Nrf2 nuclear translocation. (d) SIRT1 immunofluorescent staining (×300, bar = 100 *μ*m). Red fluorescence shows SIRT1 (row 1); blue fluorescence shows cell nuclei (row 2). (e) SIRT1 immunofluorescence intensity. (f) Relative SIRT1 activity. Data are presented as the mean ± SEM (*n* = 6 in each group). ^a/aa^ *P* < 0.05/0.01 versus the HG group, ^b/bb^ *P* < 0.05/0.01 versus the HG + SIR group, ^c/cc^ *P* < 0.05/0.01 versus the HG + SIR + HKL group, ^dd^ *P* < 0.01 versus the siSIRT1 + HG + SIR + HKL group.

Finally, we focused on the correlation between the two signaling molecules, SIRT1 and Nrf2. Both *in vivo* and *in vitro* experiments revealed that inhibiting SIRT1 signaling with EX527 or SIRT1 siRNA markedly abolished HKL-induced increase of SIRT1 expression and activity, as well as inhibited the nuclear accumulation of Nrf2. Although Nrf2 siRNA significantly abolished HKL-induced nuclear localization of Nrf2, it had little effect on SIRT1 expression or activity. Meanwhile, immunofluorescent images also showed that SIRT1 siRNA attenuated HKL-induced elevation of SIRT1 expression, whereas Nrf2 siRNA had little impact on the immunofluorescent intensity of SIRT1 (Figure 8). Taken together, these data suggested that SIRT1 might function upstream of Nrf2 signaling in mediating the cardioprotective effects of HKL against MI/R injury in T1D.

## 4. Discussion

The present study revealed several major findings: (1) HKL protected rat hearts (*in vivo*) and H9c2 cells (*in vitro*) against I/R injury in the T1D state; (2) HKL effectively reduced I/R injury-induced oxidative stress and apoptosis in T1D; and (3) the cardioprotective effects of HKL were mediated, at least in part, by activating the SIRT1-Nrf2 signaling pathway. To the best of our knowledge, this is the first report describing the cardioprotective effects and potential underlying mechanisms of HKL against MI/R injury in a T1D setting.

Over the past 30 years, the incidence of T1D has increased by several folds, making it one of the most pressing health issues worldwide [32]. Many clinical and preclinical studies have reported that individuals with T1D have excess risk of developing cardiovascular complications (e.g., myocardial infarction, ischemic stroke, heart failure, and peripheral artery disease) than do general populations [33, 34]. Furthermore, patients with T1D are more likely to suffer from lethal myocardial infarction after acute coronary syndromes and to confer a more adverse impact on the long-term prognosis than do nondiabetes patients [35]. Significant efforts have been invested to elucidate the potential mechanisms and explore rescue strategies for I/R-injured myocardium in the diabetic state. Accumulating evidence shows that ROS accumulation, contributing to the expansion of infarct size and deterioration of cardiac function, plays a critical role in MI/R-injured diabetic myocardium [36–38]. In addition, hyperglycemia, acting as an independent risk factor, worsens cardiac function, accelerates cell death, and exacerbates myocardial injury after MI/R insult via increasing ROS generation and impairing antioxidant defenses [5, 39, 40]. Consistent with these findings, data collected in this study showed that in diabetic rats, myocardial oxidative stress increased significantly after MI/R operation in comparison to that in nondiabetic rats, which might contribute to the severely impaired postischemic cardiac performance and increased cardiomyocyte apoptosis. Therefore, it is of great interest to seek effective antioxidant therapies to address MI/R-induced cardiac dysfunction in patients with T1D.

Recently, a bioactive natural compound HKL, obtained from the genus *Magnolia*, has generated much interest for its diverse biological effects, including antiarrhythmic, anti-inflammatory, and anticancer properties, without appreciable toxicity [6, 41]. Importantly, increasing attention has also been paid on its favorable effects on diabetic performance. Wu et al. demonstrated that HKL inhibited high-glucose-induced increase of inflammatory cytokine generation in human renal mesangial cells [42]. In addition, Sheu et al. reported that a low dose of HKL suppressed apoptotic signaling and cell death in high-glucose-treated human endothelial cell [43]. Many reports have confirmed that HKL is a highly effective antioxidant in inhibiting lipid peroxidation, scavenging free radicals, and protecting mitochondrial respiratory chain enzyme activity [44, 45]. Due to its highly efficient antioxidative capacity, HKL has been widely studied in protecting against I/R injury in multiple organs, for example, brain, ovary, liver, and kidney [9, 10, 46, 47]. Although Wang et al. demonstrated that, in a nondiabetic MI/R rat heart, HKL pretreatment reduced cardiac damage by reducing oxidative stress and inflammation [12], the effects and the underlying mechanisms of HKL in a diabetic MI/R model have not been studied yet. In this study, we first confirmed that HKL inhibited ROS production and MDA generation, increased the ROS scavenging activities of SOD, and depressed the expressions of gp91*^phox^* and apoptotic signaling in diabetic heart following MI/R injury, therefore attenuating myocardial oxidative stress, reducing cardiomyocyte apoptosis, and ultimately decreasing infarction size and ameliorated cardiac dysfunction. However, detailed information regarding the signaling pathways participated in HKL's protective effects against MI/R injury in diabetic state remains to be fully elucidated.

Sirtuins, belonging to the highly conserved class III histone deacetylases, act as key modulators in controlling cellular metabolic process and stress response [15]. SIRT1, the most studied mammalian sirtuin, has been extensively investigated to produce beneficial effects on glucose homeostasis and cardiovascular functions [16, 48, 49]. Numerous findings have regarded SIRT1 as a promising molecular target in abating MI/R injury in diabetes for its positive roles of regulating oxidative stress and apoptosis. Ding et al. found that SIRT1 expression was significantly decreased in diabetic myocardium, while SIRT1 overexpression markedly improved cardiac function after MI/R injury in diabetic rats via eNOS activation [50]. In addition, Yu et al. reported that impaired cardiac SIRT1 signaling further enhanced oxidative stress and apoptosis, which contributed greatly to aggravated MI/R injury in diabetic state [17, 18]. In accordance with these studies, we not only observed markedly reduced myocardial SIRT1 expression and activity in diabetic rats but also found that reperfusion injury further aggravated these effects. Numerous studies have demonstrated that SIRT1 takes an active part in the cardioprotective activities of various antioxidants, including melatonin, curcumin, and berberine [25, 27, 51]. Avtanski et al. also found that HKL supplementation dramatically upregulated the expression levels of SIRT1 and SIRT3, which promoted the beneficial effect of HKL in antagonizing the oncogenic actions of leptin in breast cancer [19], thus leading us to test HKL and SIRT1 together and to explore their potential relationship in MI/R injury. Intriguingly, our present data showed that HKL significantly enhanced SIRT1 expression and activity in both diabetic hearts and H9c2 cells following I/R operation, whereas these effects were largely abolished by EX527 or SIRT1 siRNA. In addition, inhibition of SIRT1 signaling markedly blunted the cardioprotective activity of HKL as well, indicating that SIRT1-mediated amelioration of oxidative stress and apoptosis played important parts in the protective actions of HKL.

Another novel finding of this study is that SIRT1 serves as the upstream regulator of Nrf2 signaling in mediating the cardioprotective activities of HKL. In fact, as an important antioxidant sensor, Nrf2 itself plays a crucial role in cellular antioxidant defense mechanisms. Once activated, Nrf2 translocates from the cytoplasm to the nucleus where it binds to antioxidant-response elements to promote the transcription of target genes, such as HO-1, NQO-1, SODs, catalase, glutathione *S*-transferase, *γ*-glutamylcysteine synthase, and glutathione peroxidase [52, 53]. Recently, Nrf2 signaling was demonstrated to play a crucial part in reducing MI/R injury in a diabetic state. Peake et al. found that H_2_S preconditioned the db/db diabetic mouse heart against I/R injury by activating Nrf2 signaling [54]. Duan et al. reported that butin decreased ROS-mediated apoptosis in diabetic myocardium exposed to I/R by activating Nrf2-regulated antioxidant enzymes [55]. In addition, Nrf2 signaling can be activated by HKL, as reported by Rajgopal et al., who found that HKL protects hepatocytes against oxidative stress via stimulating the Nrf2 pathway [22]. In the present study, our results showed that HKL treatment markedly improved nuclear Nrf2 accumulation and facilitated Nrf2-dependent HO-1 and NQO-1 transcription in MI/R-injured diabetic myocardium. However, SIRT1 inhibition reversed these actions, indicating that HKL might potentiate the Nrf2 signaling through SIRT1 activation. Indeed, these data were consistent with the previous studies that SIRT1 can regulate the transcription factor Nrf2 in modulating the transcription of gene encoding antioxidant enzymes to affect the cellular redox state. For example, Xue et al. demonstrated that the Nrf2/antioxidant-defense pathway participated in the neuroprotective effects of SIRT1 against focal cerebral ischemia after hyperbaric oxygen preconditioning [56]. Yu et al. also demonstrated that the Nrf-2/HO-1 signaling was enhanced via SIRT1 activation in the protective effects of diallyl trisulfide against MI/R injury [18]. Significantly, the *in vitro* experiment further proved that SIRT1 siRNA could inhibit HKL-induced nuclear Nrf2 accumulation and HO-1/NQO-1 upregulation, while Nrf2 siRNA showed little influence on SIRT1 expression and activity. Therefore, we concluded that HKL reduced myocardial oxidative damage and apoptosis, thus ameliorating MI/R injury in T1D in a SIRT1-Nrf2 signaling-dependent pathway.

Overall, our findings showed that the amelioration of I/R injury in T1D by HKL can be ascribed to its antioxidative and antiapoptotic actions. More importantly, the SIRT1-Nrf2 signaling pathway was found to play a critical role in mediating the cardioprotective effects of HKL. Our findings suggested that HKL administration might serve as an early therapeutic intervention for treating T1D patients with ischemic heart disease. Experiments using SIRT1- and Nrf2-deficient animals are warranted to further confirm these findings, and additional studies using larger animals must be conducted before HKL treatment can be used in clinical practice.

## Supplementary Materials

Supplementary Materials Figure S1: a schematic illustration of *in vivo* experimental design. T1D: type 1 diabetes; MI/R: myocardial ischemia/reperfusion; HKL: honokiol. Figure S2: the confirmation of type 1 diabetic rats establishment. (a) Nonfasting plasma glucose of control rats and type 1 diabetic rats after 7 days of streptozotocin injection. (b) Fasting plasma glucose of control rats and type 1 diabetic rats after 7 days of streptozotocin injection. (c) Intraperitoneal glucose tolerance test (IPGTT) after 7 days of streptozotocin injection. (d) Oral glucose tolerance test (OGTT) after 7 days of streptozotocin injection. Data are presented as the mean ± SEM (*n* = 8 in each group). ^+/++^ *P* < 0.05/0.01 versus the Con group. Figure S3: The effect of EX527 on diabetic hearts. (a) Representative blots. (b) Bcl-2/Bax ratio. (c) Cleaved caspase 3 expression. (d) gp91*^phox^* expression. Data are presented as the mean ± SEM (*n* = 6 in each group). Figure S4: high-glucose incubation exacerbated the oxidative stress and apoptosis of SIR-injured H9c2 cells. (a) Cellular viability. (b) Representative images of TUNEL staining (200x, bar = 200 *μ*m). The apoptotic cells were detected by TUNEL (green), and the nuclei were detected by DAPI (blue). (c) Cellular apoptotic index. (d) Representative blots. (e) SIRT1 expression. (f) Relative SIRT1 activity. (g) Bcl-2/Bax ratio. (h) Cleaved caspase 3 expression. (i) gp91*^phox^* expression. (j) Representative images of DCFH-DA staining (300x, bar = 100 *μ*m). (k) DCFH-DA intensity. Data are presented as the mean ± SEM (*n* = 6 in each group). ^ee^ *P* < 0.01 versus the no-SIR group, ^f/ff^ *P* < 0.05/0.01 versus the no-HG-treated group. Figure S5: the evaluation of the knockdown capacity of SIRT1 and Nrf2 siRNAs on SIR-injured H9c2 cells and the effect of control siRNA on HG-treated H9c2. (a) Representative blots. (b) SIRT1 expression. (c) Nrf2 expression. (d) Cellular viability. (e) Representative images of TUNEL staining (200x, bar = 200 *μ*m). The apoptotic cells were detected by TUNEL (green), and the nuclei were detected by DAPI (blue). (f) Cellular apoptotic index. (g) Representative images of DCFH-DA staining (300x, bar = 100 *μ*m). (h) DCFH-DA intensity. Data are presented as the mean ± SEM (*n* = 6 in each group). ^gg^ *P* < 0.01 versus the no-siRNA-treated group, ^hh^ *P* < 0.01 versus the Con-siRNA group.

## References

[1] Orchard T. J., Costacou T. (2010). When are type 1 diabetic patients at risk for cardiovascular disease?. *Current Diabetes Reports*.

[2] Rawshani A., Rawshani A., Franzen S. (2017). Range of risk factor levels: control, mortality, and cardiovascular outcomes in type 1 diabetes mellitus. *Circulation*.

[3] Ibanez B., Heusch G., Ovize M., Van de Werf F. (2015). Evolving therapies for myocardial ischemia/reperfusion injury. *Journal of the American College of Cardiology*.

[4] Zweier J., Talukder M. (2006). The role of oxidants and free radicals in reperfusion injury. *Cardiovascular Research*.

[5] Lubos E., Loscalzo J., Handy D. E. (2011). Glutathione peroxidase-1 in health and disease: from molecular mechanisms to therapeutic opportunities. *Antioxidants & Redox Signaling*.

[6] Fried L. E., Arbiser J. L. (2009). Honokiol, a multifunctional antiangiogenic and antitumor agent. *Antioxidants & Redox Signaling*.

[7] Pillai V. B., Samant S., Sundaresan N. R. (2015). Honokiol blocks and reverses cardiac hypertrophy in mice by activating mitochondrial Sirt3. *Nature Communications*.

[8] Prasad R., Kappes J. C., Katiyar S. K. (2016). Inhibition of NADPH oxidase 1 activity and blocking the binding of cytosolic and membrane-bound proteins by honokiol inhibit migratory potential of melanoma cells. *Oncotarget*.

[9] Yaman T. S., Agacayak E., Goruk N. Y. (2016). Protective effects of honokiol on ischemia/reperfusion injury of rat ovary: an experimental study. *Drug Design, Development and Therapy*.

[10] Yu Y., Li M., Su N. (2016). Honokiol protects against renal ischemia/reperfusion injury via the suppression of oxidative stress, iNOS, inflammation and STAT3 in rats. *Molecular Medicine Reports*.

[11] Bu Q., Liu X., Zhu Y., Liu Y., Wang Y. (2014). w007B protects brain against ischemia-reperfusion injury in rats through inhibiting inflammation, apoptosis and autophagy. *Brain Research*.

[12] Wang Y., Zhang Z. Z., Wu Y., Zhan J., He X. H., Wang Y. L. (2013). Honokiol protects rat hearts against myocardial ischemia reperfusion injury by reducing oxidative stress and inflammation. *Experimental and Therapeutic Medicine*.

[13] Atanasov A. G., Wang J. N., Gu S. P. (2013). Honokiol: a non-adipogenic PPAR*γ* agonist from nature. *Biochimica et Biophysica Acta (BBA) - General Subjects*.

[14] Hsu C. C., Chen L. F., Lin M. T., Tian Y. F. (2014). Honokiol protected against heatstroke-induced oxidative stress and inflammation in diabetic rats. *International Journal of Endocrinology*.

[15] Poulose N., Raju R. (2015). Sirtuin regulation in aging and injury. *Biochimica et Biophysica Acta (BBA) - Molecular Basis of Disease*.

[16] Zhang W., Huang Q., Zeng Z., Wu J., Zhang Y., Chen Z. (2017). Sirt1 inhibits oxidative stress in vascular endothelial cells. *Oxidative Medicine and Cellular Longevity*.

[17] Yu L., Liang H., Dong X. (2015). Reduced silent information regulator 1 signaling exacerbates myocardial ischemia-reperfusion injury in type 2 diabetic rats and the protective effect of melatonin. *Journal of Pineal Research*.

[18] Yu L., Li S., Tang X. (2017). Diallyl trisulfide ameliorates myocardial ischemia-reperfusion injury by reducing oxidative stress and endoplasmic reticulum stress-mediated apoptosis in type 1 diabetic rats: role of SIRT1 activation. *Apoptosis*.

[19] Avtanski D. B., Nagalingam A., Bonner M. Y., Arbiser J. L., Saxena N. K., Sharma D. (2015). Honokiol activates LKB1-miR-34a axis and antagonizes the oncogenic actions of leptin in breast cancer. *Oncotarget*.

[20] Do M. T., Kim H. G., Choi J. H., Jeong H. G. (2014). Metformin induces microRNA-34a to downregulate the Sirt1/Pgc-1*α*/Nrf2 pathway, leading to increased susceptibility of wild-type p53 cancer cells to oxidative stress and therapeutic agents. *Free Radical Biology & Medicine*.

[21] Huang K., Huang J., Xie X. (2013). Sirt1 resists advanced glycation end products-induced expressions of fibronectin and TGF-*β* 1 by activating the Nrf2/ARE pathway in glomerular mesangial cells. *Free Radical Biology & Medicine*.

[22] Rajgopal A., Missler S. R., Scholten J. D. (2016). *Magnolia officinalis* (Hou Po) bark extract stimulates the Nrf2-pathway in hepatocytes and protects against oxidative stress. *Journal of Ethnopharmacology*.

[23] Peschke E., Wolgast S., Bazwinsky I., Ponicke K., Muhlbauer E. (2008). Increased melatonin synthesis in pineal glands of rats in streptozotocin induced type 1 diabetes. *Journal of Pineal Research*.

[24] Kong L., Wang Y., Luo M., Tan Y., Cui W., Miao L. (2017). Prevention of streptozotocin-induced diabetic nephropathy by MG132: possible roles of Nrf2 and I*κ*B. *Oxidative Medicine and Cellular Longevity*.

[25] Yu L., Sun Y., Cheng L. (2014). Melatonin receptor-mediated protection against myocardial ischemia/reperfusion injury: role of SIRT1. *Journal of Pineal Research*.

[26] Gao E., Lei Y. H., Shang X. (2010). A novel and efficient model of coronary artery ligation and myocardial infarction in the mouse. *Circulation Research*.

[27] Yu L., Li Q., Yu B. (2016). Berberine attenuates myocardial ischemia/reperfusion injury by reducing oxidative stress and inflammation response: role of silent information regulator 1. *Oxidative Medicine and Cellular Longevity*.

[28] Yu L., Gong B., Duan W. (2017). Melatonin ameliorates myocardial ischemia/reperfusion injury in type 1 diabetic rats by preserving mitochondrial function: role of AMPK-PGC-1*α*-SIRT3 signaling. *Scientific Reports*.

[29] Yu L., Fan C., Li Z. (2017). Melatonin rescues cardiac thioredoxin system during ischemia-reperfusion injury in acute hyperglycemic state by restoring Notch1/Hes1/Akt signaling in a membrane receptor-dependent manner. *Journal of Pineal Research*.

[30] Zhang Y., Yuan D., Yao W. (2016). Hyperglycemia aggravates hepatic ischemia reperfusion injury by inducing chronic oxidative stress and inflammation. *Oxidative Medicine and Cellular Longevity*.

[31] Shen K., Feng X., Pan H., Zhang F., Xie H., Zheng S. (2017). Baicalin ameliorates experimental liver cholestasis in mice by modulation of oxidative stress, inflammation, and NRF2 transcription factor. *Oxidative Medicine and Cellular Longevity*.

[32] Rewers M., Ludvigsson J. (2016). Environmental risk factors for type 1 diabetes. *Lancet*.

[33] Orchard T. J., Costacou T., Kretowski A., Nesto R. W. (2006). Type 1 diabetes and coronary artery disease. *Diabetes Care*.

[34] Gregg E. W., Sattar N., Ali M. K. (2016). The changing face of diabetes complications. *The Lancet Diabetes & Endocrinology*.

[35] Donahoe S. M., Stewart G. C., McCabe C. H. (2007). Diabetes and mortality following acute coronary syndromes. *JAMA*.

[36] Varga Z. V., Giricz Z., Liaudet L., Hasko G., Ferdinandy P., Pacher P. (2015). Interplay of oxidative, nitrosative/nitrative stress, inflammation, cell death and autophagy in diabetic cardiomyopathy. *Biochimica et Biophysica Acta (BBA) - Molecular Basis of Disease*.

[37] Stratmann B., Worms J., Tschoepe D. (2014). Diabetic cardiomyopathy/heart failure: news regarding etiology, diagnosis, therapy. *Deutsche Medizinische Wochenschrift*.

[38] Kalogeris T., Bao Y., Korthuis R. J. (2014). Mitochondrial reactive oxygen species: a double edged sword in ischemia/reperfusion vs preconditioning. *Redox Biology*.

[39] Filippo C. D., Marfella R., Cuzzocrea S. (2005). Hyperglycemia in streptozotocin-induced diabetic rat increases infarct size associated with low levels of myocardial HO-1 during ischemia/reperfusion. *Diabetes*.

[40] Marfella R., D'Amico M., Di Filippo C. (2002). Myocardial infarction in diabetic rats: role of hyperglycaemia on infarct size and early expression of hypoxia-inducible factor 1. *Diabetologia*.

[41] Arora S., Singh S., Piazza G. A., Contreras C. M., Panyam J., Singh A. P. (2012). Honokiol: a novel natural agent for cancer prevention and therapy. *Current Molecular Medicine*.

[42] Wu J. P., Zhang W., Wu F. (2010). Honokiol: an effective inhibitor of high-glucose-induced upregulation of inflammatory cytokine production in human renal mesangial cells. *Inflammation Research*.

[43] Sheu M. L., Chiang C. K., Tsai K. S. (2008). Inhibition of NADPH oxidase-related oxidative stress-triggered signaling by honokiol suppresses high glucose-induced human endothelial cell apoptosis. *Free Radical Biology & Medicine*.

[44] Shen J. L., Man K. M., Huang P. H. (2010). Honokiol and magnolol as multifunctional antioxidative molecules for dermatologic disorders. *Molecules*.

[45] Dikalov S., Losik T., Arbiser J. L. (2008). Honokiol is a potent scavenger of superoxide and peroxyl radicals. *Biochemical Pharmacology*.

[46] Liou K. T., Shen Y. C., Chen C. F., Tsao C. M., Tsai S. K. (2003). Honokiol protects rat brain from focal cerebral ischemia-reperfusion injury by inhibiting neutrophil infiltration and reactive oxygen species production. *Brain Research*.

[47] Chiu J. H., Ho C. T., Wei Y. H., Lui W. Y., Hong C. Y. (1997). In vitro and in vivo protective effect of honokiol on rat liver from peroxidative injury. *Life Sciences*.

[48] Milne J. C., Lambert P. D., Schenk S. (2007). Small molecule activators of SIRT1 as therapeutics for the treatment of type 2 diabetes. *Nature*.

[49] D'Onofrio N., Servillo L., Balestrieri M. L. (2017). SIRT1 and SIRT6 signaling pathways in cardiovascular disease protection. *Antioxid Redox Signal*.

[50] Ding M., Lei J., Han H. (2015). SIRT1 protects against myocardial ischemia-reperfusion injury via activating eNOS in diabetic rats. *Cardiovascular Diabetology*.

[51] Yang Y., Duan W., Lin Y. (2013). SIRT1 activation by curcumin pretreatment attenuates mitochondrial oxidative damage induced by myocardial ischemia reperfusion injury. *Free Radical Biology & Medicine*.

[52] Calvert J. W., Jha S., Gundewar S. (2009). Hydrogen sulfide mediates cardioprotection through Nrf2 signaling. *Circulation Research*.

[53] Kleszczynski K., Zillikens D., Fischer T. W. (2016). Melatonin enhances mitochondrial ATP synthesis, reduces reactive oxygen species formation, and mediates translocation of the nuclear erythroid 2-related factor 2 resulting in activation of phase-2 antioxidant enzymes (*γ*-GCS, HO-1, NQO1) in ultraviolet radiation-treated normal human epidermal keratinocytes (NHEK). *Journal of Pineal Research*.

[54] Peake B. F., Nicholson C. K., Lambert J. P. (2013). Hydrogen sulfide preconditions the db/db diabetic mouse heart against ischemia-reperfusion injury by activating Nrf2 signaling in an Erk-dependent manner. *American Journal of Physiology Heart and Circulatory Physiology*.

[55] Duan J., Guan Y., Mu F. (2017). Protective effect of butin against ischemia/reperfusion-induced myocardial injury in diabetic mice: involvement of the AMPK/GSK-3*β*/Nrf2 signaling pathway. *Scientific Reports*.

[56] Xue F., Huang J. W., Ding P. Y. (2016). Nrf2/antioxidant defense pathway is involved in the neuroprotective effects of Sirt1 against focal cerebral ischemia in rats after hyperbaric oxygen preconditioning. *Behavioural Brain Research*.

