# Supplementary material for: Honokiol Ameliorates Myocardial Ischemia/Reperfusion Injury in Type 1 Diabetic Rats by Reducing Oxidative Stress and Apoptosis through Activating the SIRT1-Nrf2 Signaling Pathway
Source: Oxid Med Cell Longev. 2018 Feb 20;2018:3159801. doi: 10.1155/2018/3159801 (PMC5838504; doi:10.1155/2018/3159801)
Supplement: Supplementary Materials — Figure S1: a schematic illustration of in vivo experimental design. T1D: type 1 diabetes; MI/R: myocardial ischemia/reperfusion; HKL: honokiol. Figure S2: the confirmation of type 1 diabetic rats establishment. (a) Nonfasting plasma glucose of control rats and type 1 diabetic rats after 7 days of streptozotocin injection. (b) Fasting plasma glucose of control rats and type 1 diabetic rats after 7 days of streptozotocin injection. (c) Intraperitoneal glucose tolerance test (IPGTT) after 7 days of streptozotocin injection. (d) Oral glucose tolerance test (OGTT) after 7 days of streptozotocin injection. Data are presented as the mean ± SEM (n = 8 in each group). +/++ P < 0.05/0.01 versus the Con group. Figure S3: The effect of EX527 on diabetic hearts. (a) Representative blots. (b) Bcl-2/Bax ratio. (c) Cleaved caspase 3 expression. (d) gp91phox expression. Data are presented as the mean ± SEM (n = 6 in each group). Figure S4: high-glucose incubation exacerbated the oxidative stress and apoptosis of SIR-injured H9c2 cells. (a) Cellular viability. (b) Representative images of TUNEL staining (200x, bar = 200 μm). The apoptotic cells were detected by TUNEL (green), and the nuclei were detected by DAPI (blue). (c) Cellular apoptotic index. (d) Representative blots. (e) SIRT1 expression. (f) Relative SIRT1 activity. (g) Bcl-2/Bax ratio. (h) Cleaved caspase 3 expression. (i) gp91phox expression. (j) Representative images of DCFH-DA staining (300x, bar = 100 μm). (k) DCFH-DA intensity. Data are presented as the mean ± SEM (n = 6 in each group). ee P < 0.01 versus the no-SIR group, f/ff P < 0.05/0.01 versus the no-HG-treated group. Figure S5: the evaluation of the knockdown capacity of SIRT1 and Nrf2 siRNAs on SIR-injured H9c2 cells and the effect of control siRNA on HG-treated H9c2. (a) Representative blots. (b) SIRT1 expression. (c) Nrf2 expression. (d) Cellular viability. (e) Representative images of TUNEL staining (200x, bar = 200 μm). The apoptotic cells were detected by T [file 3159801.f1.pdf]

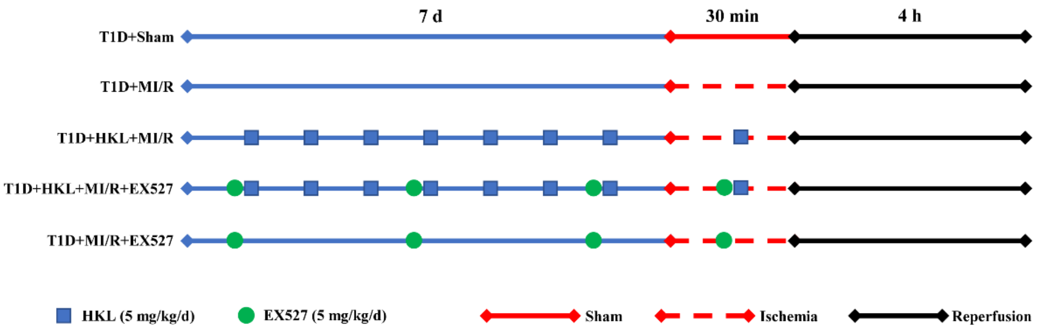

Figure S1: A schematic illustration of *in vivo* experimental design. T1D, type 1 diabetes; MI/R, myocardial ischemia-reperfusion; HKL, honokiol.

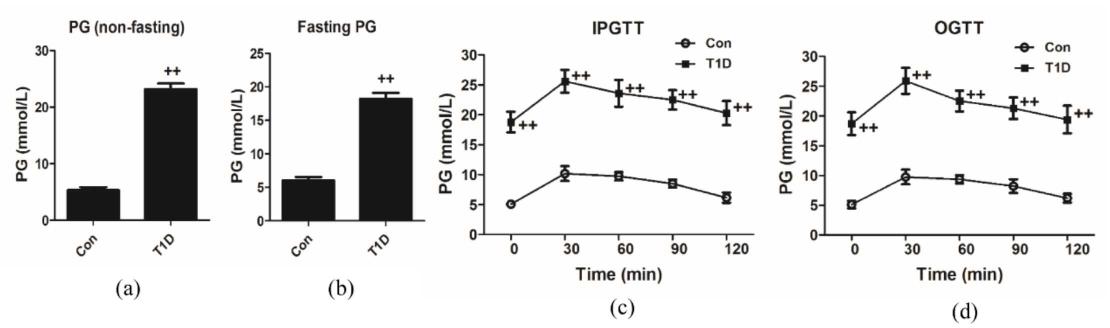

Figure S2: The confirmation of Type 1 diabetic rats establishment. (a) Non-fasting plasma glucose of control rats and type 1 diabetic rats after 7 days of streptozotocin injection. (b) Fasting plasma glucose of control rats and type 1 diabetic rats after 7 days of streptozotocin injection. (c) Intraperitoneal glucose tolerance test (IPGTT) after 7 days of streptozotocin injection. (d) Oral glucose tolerance test (OGTT) after 7 days of streptozotocin injection. Data are presented as the mean ± SEM. (n=8 in each group). $^{+/++}P < 0.05/0.01$ vs the Con group.

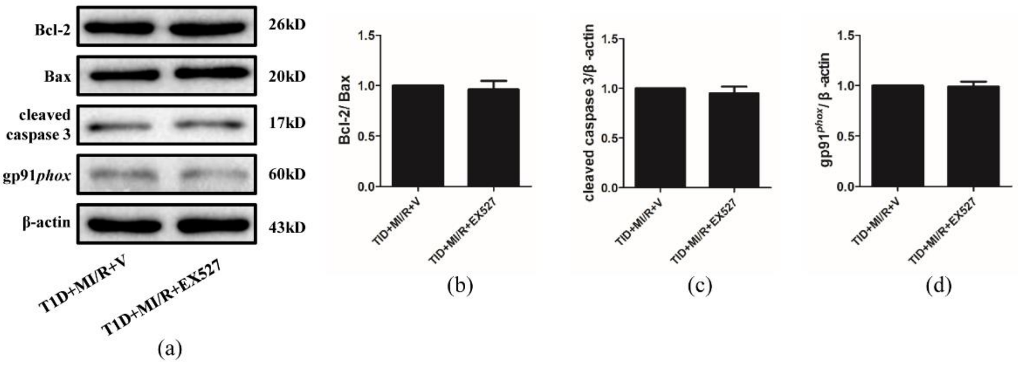

Figure S3: The effect of EX527 on diabetic hearts. (a) Representative blots. (b) Bcl2/Bax ratio. (c) cleaved caspase 3 expression. (d) gp91^{phox} expression. Data are presented as the mean ± SEM. (n=6 in each group).

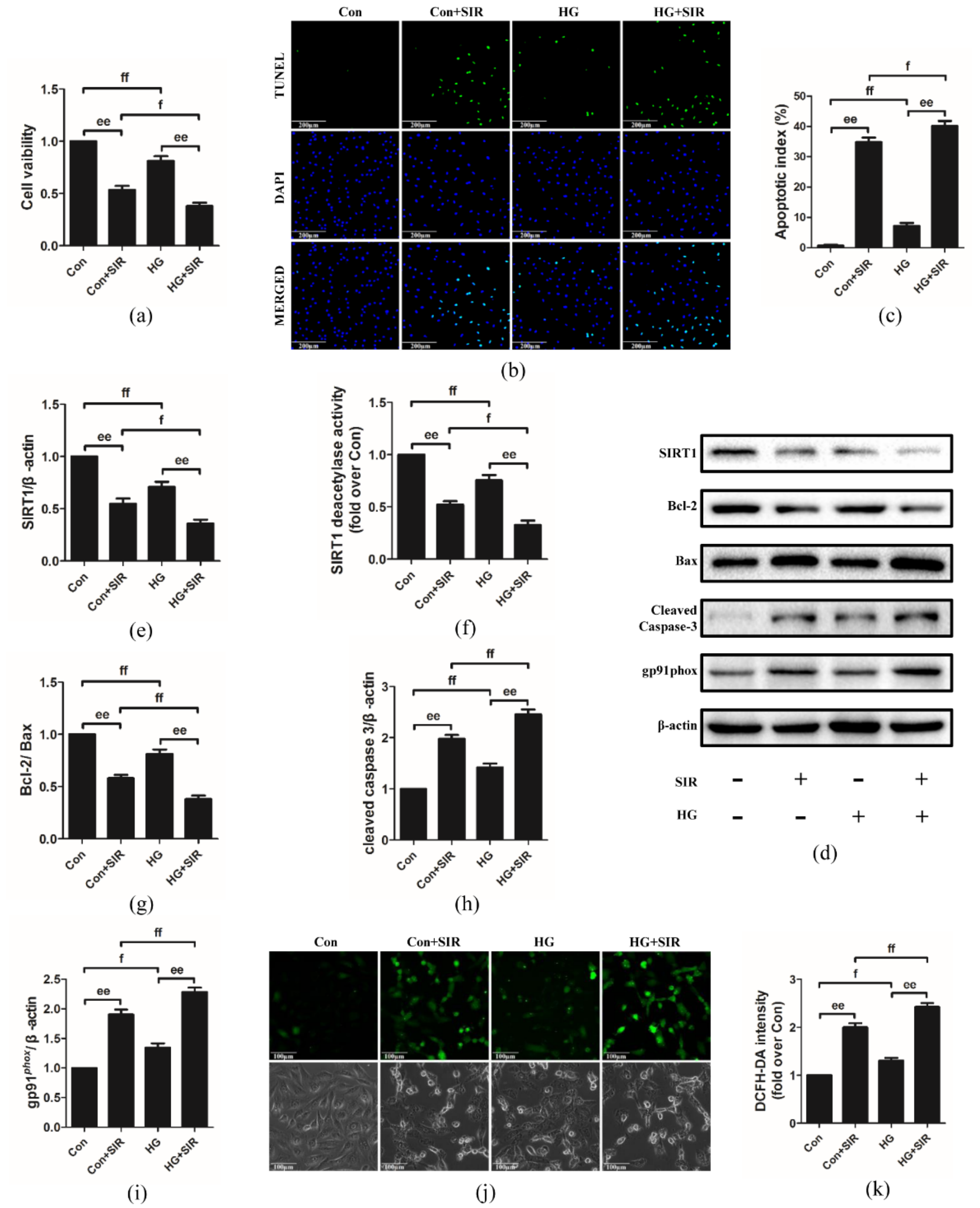

Figure S4: High-glucose incubation exacerbated the oxidative stress and apoptosis of SIR-injured H9c2 cells. (a) Cellular viability. (b) Representative images of TUNEL staining (200×, bar = 200 μm). The apoptotic cells were detected by TUNEL (green) and the nuclei were detected by DAPI (blue). (c) Cellular apoptotic index. (d) Representative blots. (e) SIRT1 expression. (f) Relative SIRT1 activity. (g) Bcl2/Bax ratio. (h) cleaved caspase 3 expression. (i) gp91^{phox} expression. (j) Representative images of DCFH-DA staining. (300×, bar = 100 μm). (k) DCFH-DA intensity. Data are presented as the mean ± SEM. (n=6 in each group). $^{ee}P < 0.01$ vs the no-SIR group, $^{f/ff}P < 0.05/0.01$ vs the no-HG-treated group.

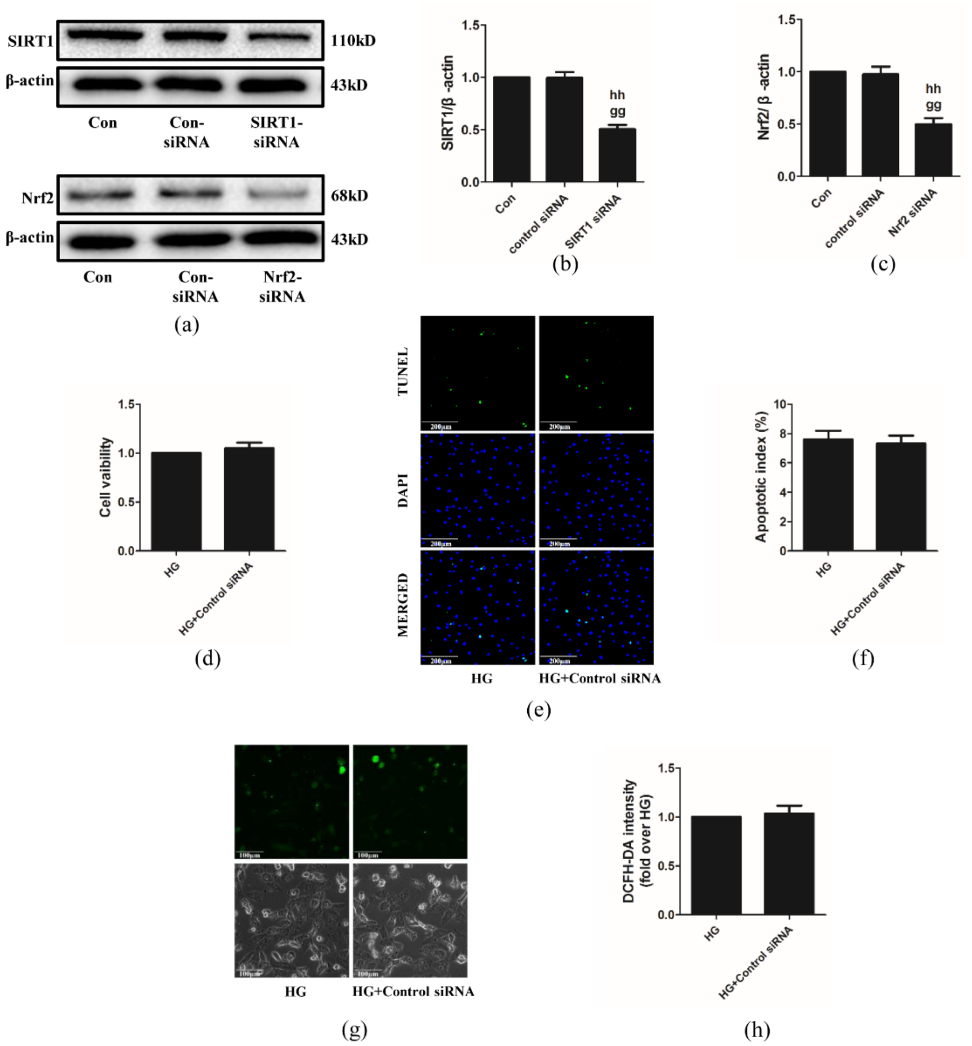

Figure S5: The evaluation of the knockdown capacity of SIRT1 and Nrf2 siRNAs on SIR-injured H9c2 cells and the effect of control siRNA on HG-treated H9c2. (a) Representative blots. (b) SIRT1 expression. (c) Nrf2 expression. (d) Cellular viability. (e) Representative images of TUNEL staining (200×, bar = 200 μm). The apoptotic cells were detected by TUNEL (green) and the nuclei were detected by DAPI (blue). (f) Cellular apoptotic index. (g) Representative images of DCFH-DA staining. (300×, bar = 100 μm). (h) DCFH-DA intensity. Data are presented as the mean ± SEM. (n=6 in each group). $^{gg}P < 0.01$ vs the no-siRNA-treated group, $^{hh}P < 0.01$ vs the Con-siRNA group.
